# An mHealth Intervention for Persons with Diabetes Type 2 Based on Acceptance and Commitment Therapy Principles: Examining Treatment Fidelity

**DOI:** 10.2196/mhealth.9942

**Published:** 2018-07-03

**Authors:** Andréa Aparecida Gonçalves Nes, Sandra van Dulmen, Espen Andreas Brembo, Hilde Eide

**Affiliations:** ^1^ Faculty of Nursing Lovisenberg Diaconal University College Oslo Norway; ^2^ Netherlands Institute for Health Services Research Utrecht Netherlands; ^3^ Department of Primary and Community Care Radboud University Medical Center Nijmegen Netherlands; ^4^ Science Centre Health and Technology Faculty of Health and Social Sciences University of South-Eastern Norway Drammen Norway

**Keywords:** diabetes mellitus type 2, Acceptance and Commitment Therapy, mobile phone, Web-based, treatment fidelity, mHealth

## Abstract

**Background:**

Web-based interventions are becoming an alternative of treatment aimed to support behavioral changes and several advantages over traditional treatments are reported. New ways of delivering an intervention may result in new challenges regarding monitoring of treatment fidelity (TF) which is essential to ensure internal and external validity. Despite the importance of the theme, only a few studies in this field are reported.

**Objective:**

To examine TF of a mobile phone delivered intervention based on Acceptance and Commitment Therapy (ACT) with electronic diaries and written situational feedback for persons with diabetes mellitus type 2, the recommendations from the Behavior Change Consortium (BCC) established by The National Institutes of Health (NHI) were applied. To analyze fidelity, they recommend 5 areas to be investigated (1) design of the study, (2) provider training, (3) delivery of treatment, (4) receipt of treatment, and (5) enactment of treatment. In the current study, these areas were examined based on the analysis of therapists’ adherence to the treatment protocol and participants’ and therapists’ experience with the intervention.

**Methods:**

To investigate the therapists’ adherence to the treatment protocol, a total of 251 written feedback text messages were divided into text segments. Qualitative thematic analyses were then performed to examine how ACT and other therapeutic processes were used in the feedback by the therapists. For the therapists’ and participants’ experience analysis, participants answered a self-reported questionnaire and participated in 2 interviews. The therapists continuously reported their experiences to the researcher responsible for the project.

**Results:**

The results show high adherence to the TF strategies 20/21 (95%) applicable items of the fidelity checklist recommended by NHI BCC were identified in the present study. Measured provider skill acquisition post-training was the only item absent in the fidelity checklist. The results also show high therapists’ adherence to the treatment protocol. All ACT processes (values, committed action, acceptance, contact with the present moment, self as context and cognitive defusion) were found in the coded text segments of the feedback in addition to communication and motivation strategies. For 336/730 (46%) of total possible text segments coded independently by 2 researchers, the interrater reliability measured by Cohen’s kappa was .85. The evaluation of participants’ and therapists’ experience with the intervention was generally positive.

**Conclusions:**

Based on the analyses of therapists’ adherence to the treatment protocol grounded by ACT-principles and participants’ and therapists’ experience with the intervention, the 5 areas of TF recommended by NHI BCC were analyzed indicating a high level of TF. These results ensure an appropriate level of internal and external validity of the study and reliable intervention results and facilitate a precise replication of this intervention concept. Web-based psychological interventions to support people with chronic conditions are becoming increasingly more common. This study supports the results from a previous study which indicated that ACT could be reliably delivered in a written web-based format.

**Trial Registration:**

ClinicalTrials.gov NCT01297049; https://clinicaltrials.gov/ct2/show/NCT01297049 (Archived by WebCite at http://www.webcitation.org/70WC4Cm4T)

## Introduction

### Overview

Internet and mobile phone technology have opened new ways to deliver health-related counseling and therapy making health care more accessible to people, especially for those who suffer from chronic illness and live far from hospitals and qualified therapists. Several advantages over face-to-face treatments such as convenience, reduced cost, and the ability to adjust plans and feedback to a participant’s individual needs are reported [1]. Treatments delivered by the internet can also enhance health literacy and health-related knowledge and support people to cope with their health problems [2]. Challenges also exist, such as technological problems and absence of face-to-face interaction. When transforming face-to-face interventions into online interventions, there is a need to evaluate if, and how, the principles of the intervention are applied in the new modus, but only a few studies in this field are reported [3]. To avoid Type I (ie, reject the null hypothesis) and Type II (ie, reject the false null hypothesis) errors, due to misinterpretation of intervention results, it is essential to plan, monitor and evaluate Treatment Fidelity (TF). The National Institutes of Health (NIH) established a Behavior Change Consortium (BCC) and defined TF as “*the methodological strategies used to monitor and enhance the reliability and validity of behavioral interventions.”* TF is a continuous evaluation of different aspects of the intervention essential to enhance reliability and validity of the independent variables. A high level of TF assures that the treatment protocol is performed as intended. Although approaches to establish TF in Web-based interventions differ from those used in interventions delivered in person, the same fidelity evaluation requirements need to be applied [4].

The NIH BCC also developed a TF protocol with the following 5 principal areas: (1) study design that builds on strategies to ensure that the stated hypotheses can adequately be tested in relation to the underlying theory and clinical processes, (2) provider training to ensure that all treatment providers have been satisfactorily trained to deliver the intervention, (3) delivery of treatment that build on strategies, for example treatment monitoring, to ensure that the intervention is delivered as intended, (4) treatment receipt strategies that involve assessing and optimizing the degree to which the participant understands and demonstrates knowledge to use treatment skills, and (5) treatment enactment that involves assessing and optimizing of the degree to which the participant applies the skills learned in treatment in daily life. [5,6]. According to the results of a study performed by Eaton and colleagues [4], the NIH BCC Treatment Fidelity Guidelines were relevant when establishing TF for online delivered interventions.

The purpose of this study was to investigate the therapists’ adherence [7] to the treatment protocol and participants’ and therapists’ experience with the mobile phone delivered intervention with electronic diaries (e-diaries) and written feedback based on Acceptance and Commitment Therapy (ACT) for persons with Diabetes Mellitus Type 2 (DMT2). Based on this investigation the 5 areas of TF recommended by NIH BCC were evaluated.

In addition to the recommendations from the NIH BCC, it is important to emphasize that an evaluation of TF of technology-based behavioral interventions carries additional challenges due to their dynamic and highly individualized nature, and elements associated with such characteristics need therefore to be evaluated as well [8]. By evaluating fidelity, it is possible to investigate if theory-based processes of the intervention are the primary mechanism of change outcomes and if they allow for precise replication and comparison amongst interventions [9].

### Acceptance and Commitment Therapy

ACT is referred to as the third generation of Cognitive Behavior Therapy (CBT). Based on Functional Contextualism Philosophy and Relational Frame Theory [10], the objective of ACT is to improve patients’ functioning and quality of life by increasing psychological flexibility, referred to as the ability to behave in accordance with life values and long-term goals also when interfering thoughts, emotions, and bodily symptoms are present [11]. Psychological flexibility can be achieved as the result of the treatment combining the 6 processes of ACT (see Table 1). These processes are overlapping and interrelated and can be introduced in different orders [10-12]. In most ACT-based treatments, exercises and metaphors are frequently used to discuss behaviors that may appear counterintuitive [13,14].

ACT shares critical features with traditional CBT approaches, such as behavior activation. Also, differences exist, as acceptance and cognitive defusion strategies are relatively unique for ACT [11,15]. The use of therapy based on theory, such as ACT, increases the chance that interventions aiming to support self-management in chronic illness will be effective. Clinical psychologists and health care professionals in related disciplines also play a vital role in the treatment of people with long-term conditions [16]. ACT has been evaluated in several randomized controlled trials for persons with DMT2 and other chronic conditions, and results confirm the utility of this approach for improving health outcomes [14,17-20].

**Table 1:** Acceptance and Commitment Therapy subprocesses.

Subprocess	Definition
Values	The deeply meaningful elements in a person's life. Values concern the ideals we have and how we want to live.
Committed action	Specific and concrete action plans guided by one's values, which also takes into account and anticipate barriers on the way.
Acceptance	Openness to experience, urges, emotions, and thoughts allowing them to come and go without a struggle.
Contact with the present moment	Being fully aware of the psychological and environmental events with openness, interest, receptiveness and without judgment.
Self as context	Allows people to be aware of psychological content without linking it to their personal identity.
Cognitive defusion	A process where people learn how to gain a perspective regarding one's thoughts, and thus manage to see their own thoughts as an outside observer and therefore avoid being affected by them.

### Study Background

The intervention concept, involving the use of mobile technology to deliver the treatment based on behavioral therapy in a written format, was developed and tested first in the Netherlands in persons with irritable bowel syndrome (IBS) [21]. Later the intervention concept was refined and tested in Norway in persons with chronic widespread pain (CWP) and DMT2, respectively [22,23]. The IBS and CWP studies were randomized controlled trials (RCTs), whereas the DMT2 was a pilot. The RCTs showed positive results concerning catastrophizing, acceptance, and illness impact at a 3 and 5-month follow-up, respectively [21,22]. The participants’ subjective experiences of the interventions in all studies were mostly positive. A summarized description of these studies is presented elsewhere [24]. Recently, a fidelity examination of the CWP study was published, showing a high level of treatment integrity [25]. We argued in this paper that the methodology also could be applied to other similar intervention concepts. Due to the significance of TF in behavior change interventions, and the need for more studies in this area, the present study aims to investigate TF of the DMT2 pilot study.

## Methods

The current study has a mixed-method design, encompassing both qualitative and quantitative data aimed to investigate the TF of a mobile phone delivered intervention with e-diaries and written situational feedback based on ACT to support persons with DMT2.

### Fidelity Strategies

In this section the strategies applied in the DMT2 pilot study aimed to ensure the 5 areas of TF recommended by NIH BCC, will be presented. These areas are: (1) study design, (2) provider training, (3) delivery of treatment, (4) treatment receipt, and (5) treatment enactment.

#### DMT2 Pilot Study Design

Table 2 shows an overview of the DMT2 pilot study protocol, giving a summary of the study described by Nes and colleagues [23,24]. As mentioned before ACT was the critical component chosen to support a behavioral change in the person with DMT2. To ensure the fidelity of the DMT2 study design, and more specifically, to ensure that stated hypotheses could adequately be tested in relation to underlying theory and clinical processes, the intervention was daily monitored by a research coordinator (HE, co-author of the current paper) with an extensive experience in teaching meditation and previous experience in writing and supervising ACT/CBT based written situational feedback aimed to support women with CWP [22]. The feedback was made available to the participants after being approved by the research coordinator. The written format of the feedback facilitated this process.

#### Provider Training

A therapist wrote the feedback text messages in the DMT2 pilot study. She had a background in health care sciences (nursing) and attended a 3-day theoretical and practical course in ACT for clinical purpose held by Professor Steven Hayes, the founder of ACT. The therapist also received training, by the research coordinator, in how to analyze the information contained in the daily e-diaries and to write the feedback text based on the e-diaries and ACT.

The training in writing of feedback text messages lasted for 1 month. The feedback texts were provided for 2 persons with DMT2 who participated in a pre-pilot during the development phase of the intervention. These feedback messages were composed in cooperation with a multidisciplinary group including, a diabetes researcher, a diabetes nurse, a communication researcher, and a nutritionist.

**Table 2:** Overview of the diabetes mellitus type 2 pilot study by Nes et al [23,24].

Protocol	Description
Aim	To develop and test the feasibility of a mobile phone delivered intervention based on ACT to support self-management in persons with diabetes mellitus type 2.
Design	This was a feasibility pilot study. The intervention lasted for 12 weeks and started with a personal instructional meeting followed by daily e-diaries and feedback via a mobile phone. At a scheduled diary-completion time and to access the feedback, the participant received a short message service (SMS) text message with a link to a secure website. In this website, the diary questions could be answered and submitted back to the server, and the available feedback message could be read. There were 4 audio files with mindfulness and relaxation exercises available on the mobile phones.
Diaries	The participants completed the e-diaries 3 times daily. The diaries included 16-19 questions chosen for supporting self-monitoring (level of blood glucose, diet, medicine, and achieved activities) and awareness of health behavior, thoughts, feelings and applied self-management strategies. Most of the questions were answered by choosing predefined alternatives or by scoring on a 6-point Likert scale. The diaries also included a comment field giving participants the opportunity to write a short personal message to the therapist.
Feedback	A therapist had immediate access to submitted diaries and used the situational information to formulate personalized feedback based on Acceptance and Commitment Therapy. The purpose of the diaries and the situational feedback was to stimulate self-management. Daily written situational feedback (except weekends) was given during the first month followed by weekly feedback during the next 2 months. A multi-disciplinary group supported the development of the feedback during the first period of the study. The therapist used information from the 3 latest submitted diaries. There was no limitation on the length of the feedback.
Therapist	A nurse that was trained in Acceptance and Commitment Therapy.
Setting and recruitment	The intention was to recruit persons with type 2 diabetes through general practitioners and to include 10-15 participants to test the feasibility of the intervention in this patient group. Because of the difficulty in recruiting participants through their general practitioners, the social network of the researchers was also informed about the project and persons were asked if they knew potential candidates. The potential candidates with type 2 diabetes that met the inclusion criteria received a letter describing the study. Those interested in participating met the responsible researcher and received additional information. After receiving complementary information, the patients who agreed to participate in the project signed an informed consent form. All patients were followed by and received standard care from their general practitioners.
Outcomes	The primary outcome was the level of glycosylated hemoglobin (HBA_1c_). This blood test shows the average level of blood sugar over the previous 2 to 3 months. This indicates how well a person with diabetes is being controlled over time. The secondary outcomes were the Audit of Diabetes Dependence Quality of Life (ADDQoL-19) [26], and Problem Areas in Diabetes (PAID) [27].
Study Sample and Data Collection	Of the 11/15 (73.3%) participants included in the study completed the intervention. The data were collected at researcher’s and general practitioner’s office. The baseline in the first meeting with the patients (T1) and immediately after 12-week intervention period (T2). The participants were interviewed twice. The first time halfway through and the second time at the end of intervention period.
Statistical Analysis	Descriptive statistics as means and frequencies were calculated using IBM SPSS version 18 statistical software. A descriptive summary of the information extracted from the interviews was made, the content was analyzed, and themes identified.
Effect	Most of the participants reported positive life style changes. The response rate to daily registration entries was good and few technical problems were encountered. The mean HBA_1c_ level the week before inclusion was 7.4% (SD 1.1%) and 6.9% (SD 0.8%) at the end of intervention. More detailed results regarding outcomes are reported elsewhere [23].
Feasibility	At the end of the intervention, the participants received a questionnaire to assess their experience with the study. The questionnaire had 5 main areas with the number of items varying from 8 to 20: (1) participation in the project (12 items), (2) use of mobile phone (20 items), (3) daily diaries (12 items), (4) the received feedback (12 items), and (5) self-management (8). The scoring range in the answers was on 5-point Likert scales from 0 ‘‘totally disagree’’ to 5 ‘‘totally agree.’’ The mean for participation in the project was 4.2 (SD 0.5), for the use of a mobile phone it was 3.3 (SD 0.2), for diaries it was 4.4 (SD 0.4), for feedback it was 4.0 (SD 0.5), and for self-management it was 3.4 (SD 0.6). The participants also answered 7 questions about the project structure. There were 2 semi-structured interviews with each participant performed.
Conclusion	The described intervention is feasible and was evaluated as supportive and meaningful. The developed mobile phone application seems a promising tool for supporting patients with type 2 diabetes to make important lifestyle changes.

#### Delivery of Treatment

To ensure that the treatment was delivered as intended, a framework with principles for the development of feedback based on ACT was created (see Multimedia Appendix 1). It is important to emphasize that the feedback text messages were tailored and formulated based on several sources of input. The most important input being the first meeting with the participant, the daily e-diaries, and ACT. Therefore, the DMT2 intervention framework (Multimedia Appendix 1) must be seen as a guide rather than a fixed plan, where the therapist had the freedom to tailor the strategies to meet individual needs best. It is also important to emphasize that the written feedback text messages were expected to contain components other than ACT that are commonly used in treatment such as communication and motivation strategies. Ultimately, the feedback text messages were intended to support and stimulate the participants’ self-management skills. The strategies applied by the therapist included positive reinforcement, information, metaphors, ACT exercises and questions aimed at encouraging mindfulness, willingness, and engagement in meaningful activities (see Multimedia Appendix 2, for examples of feedback text messages).

#### Treatment Receipt

In the first individual meeting with the potential participants of the DMT2 project, the intervention was presented and explained. For those who agreed to participate, a mobile phone and an instruction manual containing all the necessary information were provided. The researcher (AAGN) instructed each participant on how to use the mobile phone, how to complete the e-diaries and how to access and read the feedback messages. The participants also received education about the importance of identifying life values and corresponding goals according to the ACT framework. To reinforce this information, they also received a workbook with voluntary written exercises aimed to help them in this process. Contact details were included in the manual. The first week of the intervention was a run-in-period, intended to familiarize the participants with the mobile phone, the e-diaries, and the feedback. All these described practices were performed to increase the participants’ level of confidence to understand and undertake treatment-related behavioral and cognitive strategies. The fidelity of participant receipt of treatment is essential for Web-based studies due to the missing or minimal in-person contact, where many interactions rely on written communication where providers miss nonverbal cues of comprehension [8]. In the DMT2 project, participants interacted face-to-face with the researcher once in the first meeting, and twice during the data collection period including the interviews. The participants had only 1 telephone meeting with the therapist, before the start of the intervention. The purpose of this call was to establish a therapeutic alliance and to clarify the participants’ need for support. The first meeting with the researcher, mentioned before, was also aimed to receive input from the participants related to their health-related needs. By completing the e-diaries, the participants recorded daily their glucose level, physical activities, diet, and emotions. The e-diaries contained a comment field, giving the participants the opportunity to write a message directly to the therapist. This message could be a question, extra information or a compliment.

#### Treatment Enactment

Fidelity of treatment enactment is vital to ensure that participants are regularly engaging with cognitive and behavioral skills emphasized during the treatment. Cognitive skills such as value identification, goal setting, and behavioral skills as action planning were reinforced to help participants meet their goal related to blood sugar level, exercise, and diet. The main way to stimulate the participants’ adherence to these cognitive skills was through the daily e-diaries and personalized written feedback. In the evening e-diaries, the participants evaluated if the feedback was helpful regarding recommended physical activities, diet, blood sugar level and being aware of what was important for oneself. They could also indicate if the feedback was helpful or not. If the response was that the feedback was unhelpful, the therapist stimulated the participants to use the comment field to report their needs. Based on this daily report the therapist managed to help the participants with their difficulties, thereby increasing participants’ satisfaction with feedback and treatment enactment.

### Fidelity Assessment

An investigation of the therapist's adherence to the treatment protocol and participants’ and therapist's experience with the intervention was done to assess the TF. Further, a checklist, containing the list of criteria (25 items) developed by Borrelli and colleagues [28], was applied. The 25 items are divided into the 5 TF categories (Design, Training, Delivery, Receipt, and Enactment) given the possibility to evaluate all the 5 areas of fidelity recommended by the NIH BCC. The authors of this checklist defined “high treatment fidelity” as those studies that have kappa=.80 or greater proportion adherence to their checklist across all strategies.

#### Assessment of Providers’ Adherence to the Treatment Protocol

The first part of the investigation of TF was performed by assessing the therapist’s adherence to the therapy model by comparing the intended content of the feedback (see Multimedia Appendix 1) with the actual feedback given. The intention was to evaluate TF regarding the study protocol, provider training and delivery of treatment. To investigate the therapist's adherence, all data material consisting of 251 de-identified written feedback messages from the DMT2 pilot study [23] that was sent to the 11 participants who completed the intervention, was analyzed. Each participant received on average 23 feedback text messages (range 13-28). For this investigation, a coding scheme for written feedback texts developed previously [25], was refined and applied. The refinement process was divided into 3 steps: (1) a qualitative thematic analysis of the written feedback messages and identification of different codes (categories), (2) calculation of interrater reliability as one aspect of the psychometric quality of the coding scheme, and (3) coding of all feedback messages to identify how ACT principles were applied in the daily feedback text messages.

##### Qualitative Content Analysis

The feedback messages were analyzed qualitatively using a combined deductive and inductive approach [29]. The objective of the analysis was to investigate how ACT and other possible processes were applied in the written situational feedback that was given to patients with DMT2 (see Multimedia Appendix 2 for examples of feedback text messages). As a first step, the data were analyzed using a deductive approach [30,31], initiated by a priori themes representing the six ACT processes (Table 1) [32].

As a second step, the data was re-analyzed inductively using an editing organizing style to identify other therapeutic processes (ie, those not explicitly related to ACT). This iterative process involved a systematic reading of all data material where relevant observations were continuously coded and refined for further interpretation [30].

##### Interrater Reliability Assessment

Interrater reliability is a statistical measure used to examine the agreement between 2 or more people on the assignment of categories of a categorical variable [33]. It is an important measure in determining how well an application of a coding or measurement system works. The kappa statistic was chosen in this study as it is frequently used to test interrater reliability [34]. The results can, according to Cicchetti [33], range from poor to excellent (poor: kappa<.40, fair: kappa=.40 to .59, good: kappa=.60 to.74, excellent: kappa=.75 to 1.00).

To ensure the reliability of the current study, interrater reliability was assessed in 2 phases. In the first phase, 4 researchers participated in the process. They received a decoded selection of data from 5 participants of the DMT2 study where all ACT categories supposedly were represented, and the results were compared. These results are reported in an earlier publication [25], and the adaptation in the current paper was performed by AAGN, EAB and HE. The primary purpose was to check if the understanding of ACT-processes had high enough inter-coder reliability and to verify the need for refining codebook. After the inter-coder reliability control, a review process of the ACT codes was conducted. The results were compared and accordingly, the coding scheme and the codebook were adjusted, refined, and completed. The data from the 6 participants, 336/579 (58%) text segments derived from 145/251 (58%) feedback text messages, were used to calculate the final interrater reliability in the present study. The 2 researchers (AAGN &EAB) independently coded these data.

##### Analysis of all Feedback Messages

When the interrater reliability assessment produced excellent results (kappa>.75), the remaining data material (106/251, 42% feedback messages was coded by AAGN using the refined codebook.

#### Assessment of Therapists’ and Participants’ Experience With the Intervention

The second part of the fidelity investigation involved the assessment of the participant and therapist overall experience with the intervention. The purpose was to evaluate treatment receipt and treatment enactment. The participants completed a questionnaire at the end of the intervention period and took part in 2 individual interviews (see Multimedia Appendix 3, for the interview guide). The first interview was performed halfway through and the second at the end of intervention period. The therapist´s experiences were assessed and documented during several informal meetings. These informal meetings were remote (over the telephone) between the therapist and the researcher (who took notes). During the first 4 weeks, the daily telephone meetings were held (except during weekends). After the first month, weekly telephone meetings were arranged until the end of the intervention. Based on the therapist´s and participants’ experiences with the intervention being relevant to feasibility evaluation, the results regarding this investigation were presented in a previous study [23]. See Multimedia Appendix 4, for a summary of description and results of these analyses.

## Results

The first part of the results will be presented in accordance with the completed checklist of the 5 areas of TF recommended by the NIH BCC (Study design, Provider training, Treatment delivery, Receipt, and Enactment). Also, the results of the analyses that made it possible to complete the fidelity checklist as therapist's adherence to the treatment protocol (qualitative analysis, interrater reliability, and coding of all feedback messages) will be presented. The results of participants’ experience with the therapist and therapy and therapist’s experience with the intervention are summarized in the Multimedia Appendix 4.

### Fidelity Checklist

To complete the fidelity checklist, in addition to the results presented in this section, the overview of the DMT2 pilot study (Table 2) and its results (Multimedia Appendix 4), were evaluated. The purpose was to answer questions about treatment design, treatment receipt. and treatment enactment. Table 3 shows that 4/25 (16%) items of the fidelity checklist were not applicable to this study due the design DMT2 being a single arm pilot study. A total of 20/21 (95%) remaining items of the fidelity checklist were identified in the present study. These results, according to Borrelli and colleagues [28], indicate that the DMT2 study had a high level of TF.

### Qualitative Content Analysis of Therapist Feedback Text Messages

**Figure 1:**
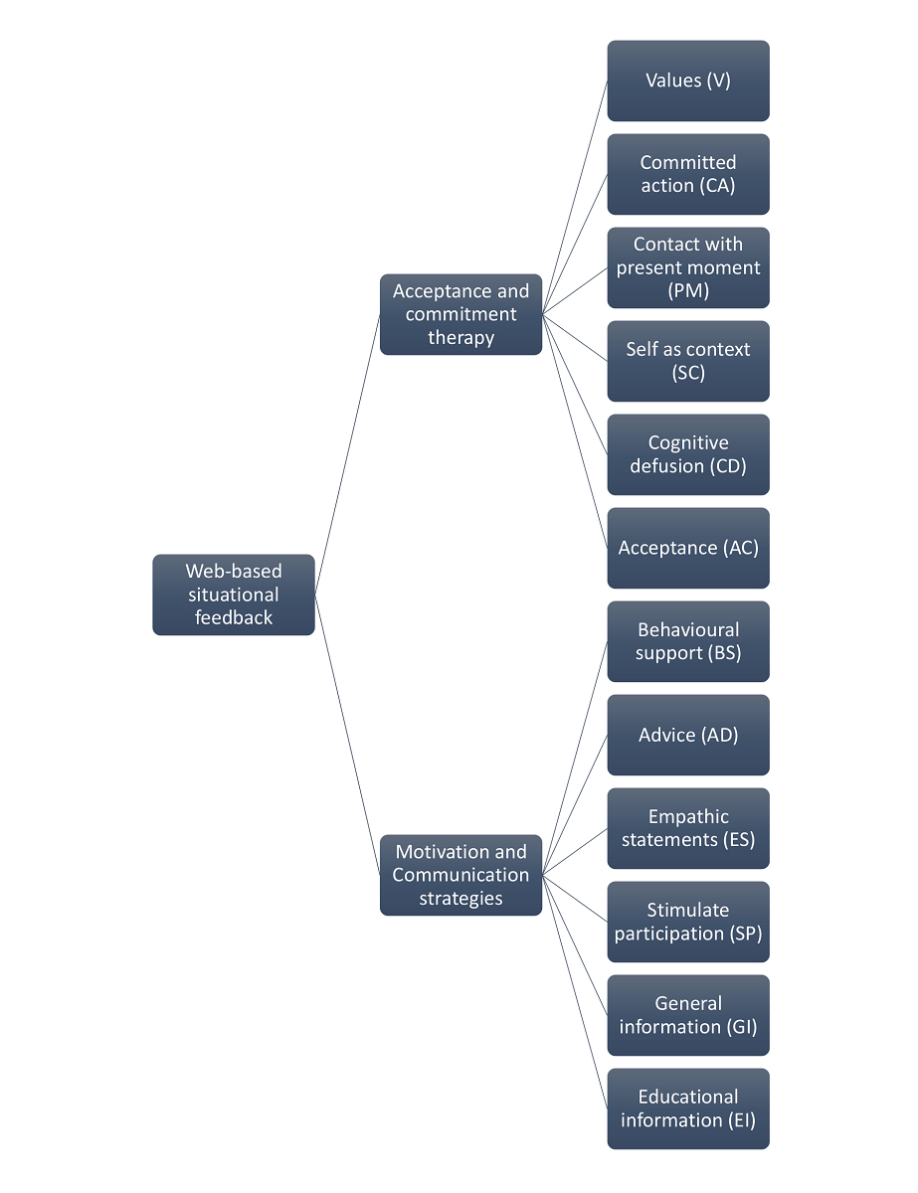
Refined coding scheme.

The qualitative analysis resulted in a refinement of a complete coding scheme that was previously developed in a comparable study [25]. The refined coding scheme included 12 codes, reflecting six ACT-processes (values, committed action, contact with the present moment, self as context, acceptance, and cognitive defusion) and 6 motivation and communication strategies (advice, empathic statements, stimulate participation, general information and educational information), see Figure 1. The ACT process, self as context, was not present in the data material of the previous pain study [25]. This previous study also identified a motivation and communication code, called creative communication, which was not present in the data of the current study.

**Table 3:** Assessment of treatment fidelity strategies developed by Borrelli and colleagues [28].

Treatment fidelity strategies			Present	Absent	Not Applicable
Treatment design					
	**Provided information about treatment dose in the intervention condition**				
		Length of contact session(s)	X	—	—
		Number of contacts	X	—	—
		Content of treatment	X	—	—
		Duration of contact over time	X	—	—
	**Provided information about treatment dose in the comparison condition**				
		Length of contact session(s)	—	—	X
		Number of contacts	—	—	X
		Content of treatment	—	—	X
		Duration of contact over time	—	—	X
	Mention of provider credentials		X	—	—
	Mention of a theoretical model or clinical guidelines on which the intervention is based		X	—	—
**Training providers**					
	Description of how providers were trained		X	—	—
	Standardized provider training		X	—	—
	Measured provider skill acquisition post training		—	X	—
	Described how provider skills maintained over time		X	—	—
**Delivery of treatment**					
	Included method to ensure that the content of the intervention was being delivered as specified		X	—	—
	Included method to ensure that the dose of the intervention was being delivered as specified		X	—	—
	Included mechanism to assess if the provider adhered to the intervention plan		X	—	—
	Assessed nonspecific treatment effects		X	—	—
	Used treatment manual		X	—	—
**Receipt of treatment**					
	Assessed subject comprehension of the intervention during the intervention period		X	—	—
	Included a strategy to improve subject comprehension of the intervention above and beyond what is included in the intervention		X	—	—
	Assessed subject’s ability to perform the intervention skills during the intervention period		X	—	—
	Included a strategy to improve subject performance of intervention skills during the intervention period		X	—	—
**Enactment of treatment skills**					
	Assessed subject performance of the intervention skills assessed in settings in which the intervention might be applied		X	—	—
	Assessed strategy to improve subject performance of the intervention skills in settings in which the intervention might be applied		X	—	—

**Table 4:** Codebook with refined codes definitions.

Processes and strategies		Code	Definition
**Acceptance and Commitment Therapy**			
	Values	V	Stimulate patient’s reflection on their own values and the values’ impact on their life. Helping the patient to identify the difference between goals and values. Stimulate awareness of gratitude and enthusiasm regarding feelings for increasing awareness of values.
	Committed action	CA	Encourage the patient to committed behavior related to their own values through reflecting on strategies related to well-planned actions, barriers and follow-up. Stimulate planning of activities in the form of value-oriented goals.
	Contact with present moment	PM	Stimulate breathing exercises for relaxation and variety in activities. Stimulate attention and awareness of internal and external experiences in the present moment.
	Cognitive defusion	CD	Stimulate awareness of thought processes instead thought content. Stimulate understanding in thought content as a result of the context in which thoughts are a product of the specific situation(s).
	Acceptance	AC	Encourage the patient to make active choices to act in accordance with their values, despite the difficult thoughts, emotions and physical sensations that are unpleasant, but which we cannot directly eliminate or reduce.
	Self as context	SC	Stimulate the patient be aware of psychological content without linking it to their personal identity, creating a sense of distance between one’s self and one’s thoughts.
**Motivation and communication**			
	Behavioral support	BS	Support the patient in the change process by recognizing the patient's willingness and efforts to change behavior. Motivate the patient in the change process through the use of praise and positive words. Provide confirmation of the patient's coping strategies. Encourage the patient to be their own supporter by practicing self-talk. Use specific records from patient diary forms in order to deliver a Message. Summarize developments during the follow-up period to emphasize key elements.
	Advise	AD	Give specific and constructive advice for appropriate behavior or to specific situation.
	Empathic statements	ES	Recognize the patient's experiences and feelings, showing empathy, understanding and respect.
	Stimulate participation	SP	Encourage patient to provide written information to ensure the most individualized follow-up.
	General information	GI	Provide information of a general, not therapeutic purpose
	Educational information	EI	Explain scientific purpose and significance related to advice, training and or intervention

The refined coding scheme was used to refine the codebook with definitions (see Table 4). The 251/251 (100%) feedback text messages, which were divided into 722/722 (100%) text segments, were coded based on the refined coding scheme and codebook. Table 5 illustrates examples of text segments and codes.

### Interrater Reliability

The interrater reliability was calculated based on 336 text segments, delivered to 6/11 (55%) included participants. The number of observed agreements was 291/336 (86.61%) of the observations). The number of agreements expected by chance was 45.9/336 (13.65%) of the observations), resulting in kappa=.85 (95% CI 0.80-0.89) and SE 0.02. Table 6 displays the distribution of the codes within the dataset.

### Analysis of All Feedback Messages

Out of the total text segments coded, 240/722 (33.2%) were ACT-consistent and 482/722 (66.8%) were coded as motivation or communication codes. All 11 participants received text segments that represented the V and CA codes. Of this 7/11 (64%) received text segments representing the CD code, 6/11 (55%) the AC code, 5/11 (45%) the PM code and 3/11 (27%) the SC code (Table 7).

**Table 5:** Example of feedback text messages divided in text segments. AD: advise; BS: behavioral support; CA: committed action; EI: education information; ES: empathetic statements; GI: general information.

Feedback Text Messages Number	Code	Text segments
3	CA	Hi. Yesterday I wrote a little about life values. If one of your life values is to achieve better health, you may set up some goals to live by according to this life value. An example of this might be that you may eat and drink according to your physician’s recommendation. The goals should not be extensive, but narrow and feasible. The mobile phone you borrowed from the project has several utilities. Have you tried to use the software for diabetes that is uploaded there? You can, for example, set up goals regarding food and drink for the day and for the week. At the end of the day, record what you have eaten and been drinking, and the system provides feedback (smile faces), depending on how you performed in relation to your own personal goals.
3	EI	The software uses the terms high and low carbs. It may be a little unclear what these terms mean, so I'm going to give you a little explanation now. High carbs are carbohydrates that are rapidly absorbed by the body and rapidly increases blood sugar. Examples of drink and food that contain a lot of carbohydrates are sugary drinks like soft drinks and pasta respectively. Low carbs are carbohydrates that are slowly absorbed by body and give a slower blood sugar rise like bread with a lot of whole grains and fiber and vegetables.
3	GI	In the manual you received from Ann in the beginning of the project you can also find this explanation with other examples.
3	ES	Feel free to write in the text field if you have any questions! Have a nice day. Regards Helen.
4	BS	Hi. It looks to me like you have a plan for the day concerning taking of your medications, blood sugar control and eat and drink as recommended. I see that you manage to achieve these goals. That’s great!
4	CA	You wrote in the middle of the day yesterday that you were at work, and that you had the opportunity to do physical exercises. Do you want to increase your activity level? Yesterday I wrote about setting up goals according to your life values. The goals shall not be to extensive, but narrow and achievable. Even when you set goals that you believe are achievable, there may be barriers that impede you from reaching them. It may be helpful to think about any barriers in relation to the goals you set up to yourself. Barriers may include time, effort or different thoughts and feelings. Time Barriers are anything that prevent many people from moving toward their goals. Can you think of any barriers that impede you from reaching your goals? Challenging work and a hectic schedule are barriers that prevent people being as physically active as they want. If you experience the same, you may reflect on what strategies you can use to reach your goals, despite the fact that time is limited.
4	GI	A long weekend is coming up and you will not get feedback until Monday. I will take a look at the diaries you will fill out during these days, to give you feedback on Tuesday.
4	AD	During this period, I would suggest that you reflect on what I have written to you in the feedback text messages so far. This because reflection can lead to awareness of things that are important to you. I wish you a nice Pentecost weekend! Regards Helen

**Table 6:** Distribution of the codes used in the interrater reliability analyses. Dashes indicate the absence of codes. AC: acceptance; AD: advise; BS: behavioral support; CA: committed action; CD: cognitive defusion; EI: education information; ES: empathetic statements; GI: general information; PM: contact with present moment; SC: self as context; SP: stimulate participation; V: values.

Code	V	CA	PM	CD	AC	SC	BS	AD	ES	SP	GI	EI	SUM
V	29	—	3	—	—	—	—	—	—	—	—	—	32
CA	3	46	—	—	—	—	—	—	—	—	—	—	49
PM	—	—	1	—	—	—	—	—	—	—	—	—	1
CD	—	—	—	9	6	—	—	—	—	—	—	2	17
AC	—	—	—	—	3	—	—	—	—	—	—	—	3
SC	—	—	—	—	—	6	—	—	—	—	—	—	6
BS	—	2	2	3	—	—	75	—	7	—	—	—	89
AD	—	1	3	—	—	—	—	29	1	—	—	—	34
ES	—	1	—	—	—	—	—	—	11	—	—	—	12
SP	—	—	—	—	—	—	—	1	—	7	—	—	8
GI	—	2	—	—	—	—	1	—	3	2	45	—	53
EI	—	—	—	1	—	—	—	—	1	—	—	30	32
SUM	32	52	9	13	9	6	76	30	23	9	45	32	336

**Table 7:** Overview of all data material coded. Dashes indicate the absence of codes. AC: acceptance; AD: advise; BS: behavioral support; CA: committed action; CD: cognitive defusion; EI: education information; ES: empathetic statements; GI: general information; P: participant; PM: contact with present moment; SC: self as context; SP: stimulate participation; TF: total feedback messages; TTS: total of text segments; V: values.

P	TF	V	CA	PM	CD	SC	AC	BS	AD	ES	SP	CC	GI	EI	TTS
1	21	6	14	—	2	—	—	14	7	4	2	—	11	2	62
2	25	12	22	3	—	—	—	29	8	27	7	—	15	5	128
3	15	3	7	—	2	—	2	7	5	2	6	—	9	6	49
4	18	7	13	—	—	—	—	14	2	2	9	—	12	3	62
5	27	4	12	—	3	—	2	17	6	5	6	—	6	6	69
6	26	6	14	—	3	—	4	18	10	—	1	—	6	2	64
7	13	2	4	—	—	—	—	12	2	7	—	—	5	7	39
8	27	5	12	2	—	1	2	14	3	2	3	—	5	10	59
9	24	6	4	2	3	—	3	9	12	9	3	—	8	7	66
10	28	7	10	2	3	1	—	15	3	4	1	—	6	8	60
11	27	6	10	2	3	4	3	15	4	4	1	—	6	6	64
SUM	251	64	124	11	19	6	16	164	62	66	39	—	89	62	722

## Discussion

### Principal Findings

To increase treatment integrity and consequently the validity of behavioral interventions and implementation, assessment of TF is necessary. The methods of implementing and evaluating TF need to be adjusted for Web-based technology interventions due to the variability within and across these interventions [6]. In this DMT2 pilot study, the 5 areas of TF recommended by NIH BCC were applied. Another TF framework that could have been applied in this study is described by Dabbs and colleagues [8]. Their TF framework was developed for Web-based technology-interventions and also recommends the evaluation of participant’ acceptance of the intervention using the Technology Acceptance Model scales [35]. The Acceptance Model scales were not applied in the DMT2 pilot study. Still, owing to the importance to investigate the technology acceptance for the fidelity evaluation in mHealth interventions [8], several questions about this theme were included in the self-reported questionnaire developed to evaluate the DMT2 pilot study [23]. The positive results regarding participants’ acceptance of the used technology reported in the DMT2 pilot study may have contributed to the positive results of the current study regarding treatment receipt and enactment.

The evaluation of the TF, in the current study, was based on the analysis of the delivered feedback messages and by assessing participants’ and therapist's experience with the intervention. The analysis of the 251/251 (100%) written feedback messages was possible by dividing them into 722/722 (100%) text segments and subsequent coding of each segment. A comparison of the refined coding scheme and codebook with the ones developed in a previous study [25], revealed 2 differences. The first was the presence of the self as context and the second was the absence of creative communication. This result confirms that ACT processes can successfully be delivered in a written format, including self as context. Although the ACT process self as context was not present within the provided feedback in the CWP-study, it was included as a component in the e-diaries. The use of communication strategies is a necessity in all behavior change interventions. Motivation elements contribute, along with support, to stimulate the participants to complete the intervention. In the present study, 6/7 (85.7%) codes (compared with the previous study) [25] related to motivation/communication strategies were found after the qualitative content analysis. The use of creative communication (absent in the present study) can be perceived as a therapist style of communication and may not influence the results of the fidelity analysis.

Analysis of texts has become a valuable research tool in numerous areas and coding is a crucial part of these analyses. For establishing TF, a prerequisite for such analysis is to ensure firm consistency between the text and the coding. Quality control of this consistency is essential to relate the results to the treatment [36]. In the present study, we achieved an excellent level of interrater reliability. This result confirmed that the coding scheme and the codebook were reliable tools for analyzing all feedback messages making further analyses possible. In short, the results of the feedback analyses showed that all participants received the text segments coded as “values” (V) and “committed action” (CA). These two ACT-processes are essential to build up participants' understanding of the therapeutic process. Not all participants received text segments representing all ACT codes. It is important to emphasize that although the development of feedback messages was based on ACT, this does not imply that all ACT processes are required for the treatment of each participant.

The excellent representation of the different ACT codes in the feedback messages (Table 7) confirms that these were developed according to the ACT framework and principles (Multimedia Appendix 1). This means that the intervention was delivered according to the chosen theory and as intended, indicating that the TF regarding study design, provider training, and treatment delivery was effective.

The use of ACT-elements may have contributed to the positive results achieved in the DMT2 pilot study [23]. As the impact of Web-based interventions increases, theory is used more extensively [16]. According to Riley (2011), theories may need to be revised to fit the new format of Web-based and mobile interventions [37]. Our experience is that applying ACT in a written format is well suited for Web-based interventions and enables and facilitates the analysis regarding TF. A total of 806/2231 (36%) of the coded text-segments in the previous study [25] were ACT codes against 240/722 (33%) in the present study. These results can indicate that communication and motivation strategies are essential elements of the feedback messages to deliver ACTs therapeutic processes in a written format. This hypothesis needs further investigation.

Despite the positive effects achieved in ACT-based interventions, it is common for the achieved effects to diminish over time, with the return of old cognitions and activity patterns [38,39]. Other studies also show that the long-term effect of cognitive behavioral therapies is generally limited [40,41]. This may indicate a need for more continuity and a longer duration of the intervention to support self-management in people with chronic illness. The results regarding participants’ (self-reported questionnaire and interviews) and therapist's experience with the project indicate that the therapist was capable and qualified and that the participants acquired knowledge regarding their diabetes, treatment skills and how to apply this knowledge in their daily life (treatment receipt and enactment). Despite the positive results, the use of a self-reported questionnaire to assess the therapist’s experience with the project would have been more reliable. The fidelity evaluation was done by applying the checklist developed by Borelli and colleges and showed a high level of TF. This fidelity assessing tool was based on the 5 areas of TF recommended by NIH BCC being suitable for this study purpose supporting the findings of a previous study [4].

TF in online delivered intervention is an emerging area of research [42]. Therefore, methods of evaluating TF adapted to the different kinds of online interventions is needed. In the present study, the reliable and precise evaluation of therapist's adherence was possible due to a coding scheme and a codebook developed in a previous study [25]. A similar method was applied in a recent study aimed to develop and evaluate a scale for assessing therapist fidelity in Web-delivered cognitive behavior therapy [42]. The concept of using a coding system to evaluate a behavior intervention delivered in a written format provides input for future studies.

### Strengths and Limitation

The coding scheme applied in the present study showed to be reliable and valid to investigate several areas of TF. In this study, a coding scheme and a codebook for ACT-oriented Web-based personal feedback developed in a previous study were refined maintaining the high level of interrater reliability. Results based on the refined coding scheme showed that the feedback provided complied with core ACT principles. The reliability of the investigation of the therapists’ competence could be improved by the development of self-reported questionnaires to be filled in by the therapists.

The fact that the DMT2 intervention required e-diaries 3 times daily could be perceived as potential burden for the participants. The high percentage of dropouts 4/15 (26.7%) indicates that participants believe this as well. The DMT2 pilot study percentage of dropouts is equivalent to results that are reported in a comparable study [22]. However, it is important to emphasize that the participants who left the DMT2 pilot study never started the intervention. Despite being favorable to the intervention at the first meeting, they believed that to participate in the project would be too time-consuming, especially when completing three e-diaries a day. In contrast, all participants who started completed the intervention. Their experience with completing the diaries was assessed and reported elsewhere [23]. Only 2/11 (18.2%) participants would prefer fewer e-diaries and questions. A comparable study reported a higher level of participants burden, 3/6 (50%) participants considered the number of questions in each diary to be too high [43]. The number of questions in the DMT2 was fewer compared to this similar study, and this can explain the better results. However, it is essential to bear in mind the need to reduce the burden on the respondents as much as possible, as it has an adverse effect on the respondents’ motivation and thereby impacts negatively on their response quality and TF [44]. It is essential to identify the ideal number of diaries and questions delivered per day to decrease the participant burden and subsequently reduce the risk of dropouts. Furthermore, it is important to gain an in-depth understanding of participants’ reasons for early withdrawal. We encourage other researchers to consider this when planning large-scale studies requiring a large sample size and TF evaluations.

Regarding providers burden, the DMT2 pilot study revealed that it was time-consuming for the therapist to give feedback. The system did not show historical information as a summary, and it was necessary to navigate through several pages to get needed information. Also, the therapist had to look at the feedback history to avoid repeating information. After the first month, when a feedback bank was created, the time used to formulate the feedback text messages was reduced to 15–20 min, a level that providers reported as suitable [23].

Current Web-based interventions, which utilize written personalized therapeutic feedback tend to be time-consuming for the health personnel involved [22,23]. The possibilities within information technology regarding automation of parts of the feedback messages should, therefore, be explored [25]. As discussed later in the practical implication section, the automation of the feedback messages using algorithms would be possible to secure the TF in these kinds of interventions regarding study design, monitoring and treatment delivery. The potential to use artificial intelligence to analyze the relation between diaries, feedback and results continuously, is a fascinating area for future research.

The results of the present study showed that provider training was done according to the NIH BCC recommendations. The small number of participants (n=11) and the therapist (n=1), enabled the training and monitoring of the therapist as described in the method section. For a large-scale intervention, a systematic TF project should include standardized therapist training program with a focus on the appropriate diagnosis and the appropriate treatment approach. The standardization allows for the specific skills required for intervention delivery to be improved and accentuated within providers regardless of inherent differences [6]. The therapists should also attain a certification after a comprehensive training process. All the therapists should have a bachelor degree in health care like medicine, psychology, physiotherapy or nursing because of the physical and mental symptoms of the participants’ disease and the nature of the intervention (formulation of feedback messages based on information from the e-diaries and ACT) [24]. The monitoring process could be done by midterm randomized audits.

As shown in Table 2, the results of the DMT2 pilot study were positive. As expected, due to the small sample size the results were not statistically significant. Because the results of the present study indicate a high level of TF it is possible to assume the DMT2 pilot intervention results were reliable. This again provides a firm basis to recommend a RCT. This can provide a more qualified answer regarding whether the offered intervention will help more people with DMT2 to achieve self-management and increase their quality of life.

As mentioned in the current paper, by evaluating fidelity, it is possible to investigate if theory-based processes of the intervention are the primary mechanism of change outcomes and if they allow for precise replication and comparison amongst interventions.

The main contribution of this paper to the use of Web-based mobile technology in health literature regarding TF is the method of analyzing therapist’s adherence to the treatment protocol. The method presented in the current study allows more precise results when compared to the methods that are commonly used in TF studies based on observation and interpretation from another professional. Results that are based on the evaluation of people may carry several biases (ie, personal experience, humor, knowledge) that may influence the results.

With the method used in the present study, it was possible to demonstrate that the intervention based on ACT was performed as proposed, confirming that the chosen theory was the primary mechanism of change outcomes. However, the use of the method of evaluation of therapists’ adherence proposed in the current study is limited to a treatment delivered in a written format. To the best of our knowledge, this is the second study that analyses therapists’ adherence to the study protocol using this method.

### Practice Implications

A reliable and valid coding system as defined in this study is essential for exploring therapeutic change processes in this type of mobile phone-delivered interventions. The coding scheme has the potential to lay a foundation for the automation of feedback messages, together with a bank or database of these messages. Automatic feedback could be generated from a database and combined with individualized feedback if the diaries indicate this to be required. The intervention could then be developed as an application for mobile phones, reducing therapist time and costs. Making such an application available as support for clinical practices and in maintenance treatments would help treat people who do not have easy access to health care services. The use of new and innovative technology to make this kind of behavior change interventions more effective, while still taking care of the patients’ individual needs, suggests an exciting future area of research. The first step would be to develop and test the concept of automation in an RCT. In a further development of the intervention, it would be interesting to explore the effects of using more technologically advanced capabilities to gather rich and complex data. This could include sensors to measure activity levels and context-triggered diary questions [37,45]. Also, the automated feedback on registered data could be provided in progress charts, graphs, and summaries. Educational information could be given by interactive animations or videos [46].

### Conclusion

Web-based interventions are becoming increasingly popular and appear to be an essential and cost-effective supplement to everyday health care in the near future. The evaluation of TF is essential to interpret the results of interventions and its underlying working mechanisms. A different methodology is used to deliver interventions via the internet. To achieve TF, the evaluation methods must be adjusted to each kind of online treatment. By doing this, the TF in Web-based interventions can be maintained and monitored reliably.

## Appendix

Multimedia Appendix 1 Framework and principles for development of feedback based on ACT.

Multimedia Appendix 2 Example of a sequence of feedback from the first week of the project

Multimedia Appendix 3 Interview guide for evaluation of the DMT2 pilot study.

Multimedia Appendix 4 Assessment of provider’s and participants’ evaluation of intervention.

## Abbreviations

AC: acceptance
AD: advise
ACT: Acceptance and Commitment Therapy
ADDQoL-19: Audit of Diabetes Dependence Quality of Life
BCC: Behavior Change Consortium
BS: behavioral support
CA: committed action
CD: cognitive defusion
CBT: Cognitive Behavior Therapy
CWP: chronic widespread pain
DMT2: diabetes mellitus type 2
e-diaries: electronic diaries
EI: education information
ES: empathetic statements
GI: general information
HBA_1c_: glycosylated hemoglobin
IBS: irritable bowel syndrome
NIH: National Institutes of Health
PAID: Problem Areas in Diabetes
PM: contact with present moment
RCT: randomized controlled trial
SC: self as context
SMS: short message service
SP: stimulate participation
TF: treatment fidelity
V: values
