# Supplementary material for: An mHealth Intervention for Persons with Diabetes Type 2 Based on Acceptance and Commitment Therapy Principles: Examining Treatment Fidelity
Source: JMIR Mhealth Uhealth. 2018 Jul 3;6(7):e151. doi: 10.2196/mhealth.9942 (PMC6053615; doi:10.2196/mhealth.9942)
Supplement: Multimedia Appendix 1 [file mhealth_v6i7e151_app1.pdf]

Multimedia Appendix 1. Framework and principles for development of feedback based on Acceptance and Commitment Therapy

	Day 1	Day 2	Day 3	Day 4	Day 5
Week 1	Welcome to the project	Encourage the identification of values related to life and health	Stimulation of value reflection connected to life values and their role in relation to behavioral change	Encourage the awareness of the behavior guided by life and health values	Encourage the behaviour guided by life and health values
Week 2	Reminder of values	Identification of difference between goals and values related to health values	Understanding of goals balance to achieve the goals related to health values	Stimulation of goal achievement	Reinforcement of goal balance to achieve the desirable goals
Week 3	Reminder of goals	Identification of the barrier related to goal achievement	Identification of strategies to defeat the barriers	Working with strategies to defeat the barriers	Reinforcement of strategies to defeat the barriers
Week 4	Reminder of strategies to defeat the barriers	Understanding how thoughts and feelings influences behavior	Working with awareness and acceptance strategies connected to thoughts and feelings to defeat negative behaviors	Working with willingness to act in accordance with health-related values	Reinforcement of willingness to act in accordance with health-related values
Week 5 and 9					Reminder of values and goals. See week 1 and 2
Week 6 and 10					Reminder of strategies to defeat the barriers. Se week 3
Week 7 and 11					Reminder of awareness and acceptance strategies connected to thoughts and feelings to defeat negative behaviors. See week 4
Week 8 and 12					Reminder of willingness to act in accordance with health-related values. See week 4
