# Supplementary material for: An mHealth Intervention for Persons with Diabetes Type 2 Based on Acceptance and Commitment Therapy Principles: Examining Treatment Fidelity
Source: JMIR Mhealth Uhealth. 2018 Jul 3;6(7):e151. doi: 10.2196/mhealth.9942 (PMC6053615; doi:10.2196/mhealth.9942)
Supplement: Multimedia Appendix 2 [file mhealth_v6i7e151_app2.pdf]

Multimedia Appendix 2. Example of a sequence of feedback from the first week of the project

Day	*Feedback message*
1	First of all, I would like to thank you for a nice telephone meeting on Friday. Welcome to this project! The fact that you want to join us in this project shows that you want to do something to cope better with your diabetes. I can see, by analyzing your diaries, that you have started very well! You manage well the to handle the smartphone and to complete the e-diaries. This is good! From now you will receive daily feedback from me delivered by the smartphone. I see that you have registered that you are not always satisfied with your food and drink intake, even though you plan to eat and drink as recommended. Perhaps this is connected to what we were talking about on Friday? That it can be difficult to avoid sweet things like cakes and snobs? Especially on weekends that you need to work? Eat behavior is an area that many people experience as challenging. I hope we can work on this in the project period and that you can became more satisfied with your eating behavior. You told me that you bought a new bike and that you plan to take a ride in the field that you have near your home!? It looks like you've had a busy weekend at work, so you might not have had time to test your bike yet?! It seems to me that you are good at living an active life, I think you should be proud of yourself! Have a nice day! Regards Marie**.**
2	Hello. So great you have been on a bike ride! Did the bike stand the expectations? What's great that you set goals that you can accomplish! Does this also apply to the diet? You registered in the evening that you are quite satisfied with what you have eaten / been drinking. Does this indicate that you have achieved the goals you had set? Please write something in the text box about this. You registered positive feelings in the evening, so it seems like you were happy with the day. I see that you registered that the feedback made you aware of what is important to you. I would like to take this further. You have been given a workbook where there are some values exercises in the beginning, have you looked at this? I recommend you spend time working on this in the coming days. You can choose an area where you want to work. You decide. Think through what values you have in relation to this area. What is important to you? Write it down in the workbook. Only you who knows what matters and is meaningful to yourself. Think about how you live and act in relation to your life values. This is a topic that I will come back to. Have an enjoyable day! Regards Marie
3	Hello. Last night, you registered that you were satisfied with your food and drink intake and that you have eaten and been drinking as recommended. Congratulations! I can see that from the morning you planned an intense session with physical activity. It seems that you did not complete your plan from yesterday because you registered that you weren't satisfied with your activity level? On another hand, in the evening you wrote that you've done pleasurable activities, and that the feedback has helped you to be conscious of what's important to you. Filling up time with activities that bring you joy is very important and it's great that you spend time on this! Yesterday I introduced you to values, have you had time to think about it? I would like to continue to write about this theme. Values are something you choose and feel meaningful. Values can be a kind of compass controlling the direction of your actions towards a meaningful life. Imagine what you want, no matter how unrealistic you feel it is right now. I suggest you note down what you think about your values either in the workbook or in a diary. Once you've identified your values, set up some goals that can help you act in line with these. The goals should not be big but small and achievable. This strategy leads you to take small steps towards your values. Even though every step you take in the right direction may seem small, together and over time it will be very important to your life. I will write more on this later. Have an enjoyable day. Regards Marie.
4	Hello. You registered last night that you were not so happy with your activity level. It appears that you have been working a lot in the last few days. In the days you work, I see that you do not register any physical activity. You told me you use to ride bicycle to and from work, do you still do that? If so, then I would say that you are doing physical activity in the days you are working. Do you want to further increase your activity level? What do you think about this yourself? I don't know your experience, but many people find it hard to get time for performing physical activities in a busy day. To help getting some extra energy, I will recommend you relaxation exercises for you. Your smartphone contains audio files that guide the performance of such exercises. For example, you can try the one called "Relaxation Exercise", maybe this is something for you? It is also described in the instruction manual how to find the audio files with these exercises. I see that you wrote yesterday that you are having trouble typing in the text box, remember that you can call the researcher if you need help with your smartphone. Then you can continue to write if you want to convey something to me! Have a nice evening. Regards Marie
5	Hello. You registered last night that you were happy with your activity level, at least a little. Does it mean that you have taken the bike ride to and from work?! If I do not remember completely wrong, you worked this last weekend, didn't you? Then you may have free this weekend? We humans are "animals", and much we do in everyday life can easily be automated. Does this match you? How about planning something nice this weekend that you usually do not have time for? This is just a suggestion and I do not know if this suits you. Earlier this week I wrote about values. If time goes by, feel free to write a little about your values in the workbook you received from the researcher. This weekend you will not receive feedback from me. I'll see the forms you fill out this weekend to give you feedback on Monday. Wish you a good weekend! Regards Marie
