# Supplementary material for: An mHealth Intervention for Persons with Diabetes Type 2 Based on Acceptance and Commitment Therapy Principles: Examining Treatment Fidelity
Source: JMIR Mhealth Uhealth. 2018 Jul 3;6(7):e151. doi: 10.2196/mhealth.9942 (PMC6053615; doi:10.2196/mhealth.9942)
Supplement: Multimedia Appendix 3 [file mhealth_v6i7e151_app3.pdf]

Multimedia Appendix 3. Interview guide for evaluation of the Diabetes Mellitus Type 2 pilot study

Participant: Date:

1. How has this project been for you so far?

2. Tell me about your experience of the diary.

3. Tell me about your experience of the received feedback from the therapist.

4. Tell me about your experience using the smartphone.

5. How has your participation in the project contributed to ...

- ... your own perception of what matters to you?
- ... the identification of your values?
- ... health related goals in line with your values?
- ... identification of barriers that hinder the implementation of the goals?
- ... identification of solutions to the obstacles?
- ... your activities being in line with your values?
- ... the acceptance of thoughts and feelings that you have?
- ... your mastering of what you expect?

6. How do you want to be supported further in the project?

7. Do you have suggestions for improvements?
