# Supplementary material for: An mHealth Intervention for Persons with Diabetes Type 2 Based on Acceptance and Commitment Therapy Principles: Examining Treatment Fidelity
Source: JMIR Mhealth Uhealth. 2018 Jul 3;6(7):e151. doi: 10.2196/mhealth.9942 (PMC6053615; doi:10.2196/mhealth.9942)
Supplement: Multimedia Appendix 4 [file mhealth_v6i7e151_app4.pdf]

Multimedia Appendix 4. Assessment of provider's and participants' evaluation of intervention

Evaluation questionnaire	
Methods	Results
At the end of the study, the participants received a questionnaire to assess participants' experience with the e-diaries, feedback messages and the use of technology. The questionnaire had five main areas with the number of items varying from eight to twenty: (a) participation in the project (12 items), (b) use of smartphone (20 items), (c) daily diaries (12 items), (d) the received feedbacks (12 items), and (e) self-management (8). with score answers ranging from one (totally agree) to five (totally disagree). The participants have also answered seven questions about the project and structure	The participants were satisfied with the project and most of them perceived this experience as supportive, meaningful and motivating. The use of a smartphone was challenging for some participants, but most them found the phones user friendly. The main problems were the use of touch display (unusual, illogical with small letters) and the size of the telephone (too big). The diary response rate ranged from 21% to 97% with a mean of 68%. Most of the participants reported the e-diaries as a positive experience and judged it questions to be easy, inspiring and useful. Most of the participants reported positive experience with the content of the feedback messages. The feedback helped them to better manage their diabetes by reinforcing favorable coping strategies. Most participants were able to follow the advices related to physical and relaxation exercises. The feedback also encouraged the participants to follow their diet plan, check their blood glucose levels regularly and take their medication at the correct time and dosage. Just one participant reported no changes in the advising areas and three did not feel motivated to do relaxation exercises.

Interviews	
Methods	Results
To get an in depth understanding of participants' experiences, they were interviewed twice; halfway through and after the completion of the study. In these semi-structured interviews (see interview guide in Multimedia Appendix 3), the patients shared their experience with the intervention and where encouraged to provided suggestions for future improvements. The interviews were audio-recorded and transcribed	One of the participants that completed the intervention was interviewed only once (at the end of the intervention), the others, as mentioned before were interviewed twice. The feedback messages were experienced as personal and relevant to the patients' current situation. The patients reported that the feedback helped them to define their own goals based on health values, identify barriers related to goals achievement and develop strategies to overcome the barriers. They reported that they became committed to their values and goals and most of them were willing to change their life style to achieve a good health status. The patients would have liked to receive more information about diet, as they reported that this theme was difficult to deal with. The stimuli to perform physical activities helped the patients to be more active. Most of the participants would have liked to have had an overview of all feedback messages to read again when needed.

Therapist's experience with the treatment	
Methods	Results
The researcher AAGN had daily contact with the therapist with the purpose of hearing about their experiences regarding the e-diaries interpretation, the feedback formulation and supervision. The therapist also shared her experiences regarding participants treatment receipt and enactment. The researcher took notes of this information, add with own experience and summarized it with the purpose of improving the intervention increasing the treatment fidelity. These were informal contacts and the conversations were made by telephone without a guideline and time of duration.	The therapist analyzed the participants' answers in the three last diary entries before writing a feedback. After identifying important issues in the e-diaries, the therapists wrote the feedback based on these and following ACT-principles. In addition, the therapists had to look at the feedback history to avoid repeating information. A feedback ''bank'' was developed by the therapists with the contribution from other health professionals; a researcher in therapeutic communication, a researcher in diabetes care, a nutritionist, and one other nurse researcher with clinical experience. The feedback messages were approved by the supervisor before being sent to the participant. The therapist expected that the participants had sufficient knowledge of diabetes and diabetes treatment. This was not the case. The intention with the feedback was to stimulate self-management, but it was often necessary to provide basic information about diabetes first. Therefore, educational information as how diet, hydration, medicine and exercises influence the self-management of diabetes were given in the daily diaries. These kinds of information were tailored to the participants needs when identified in the e-daily diaries.
